# Respiratory viral infections in professional ice hockey teams—a one-season observational study

**DOI:** 10.3389/fspor.2026.1832991

**Published:** 2026-06-23

**Authors:** Wilma Grönroos, Maarit Valtonen, Niklas Lindblad, Timo Hänninen, Raakel Luoto, Olli J. Heinonen, Matti Waris, Minna Paloniemi, Tapio Seiskari, Olli Ruuskanen

**Affiliations:** 1 Unit for Health and Physical Activity, Paavo Nurmi Centre, University of Turku, Turku, Finland; 2 Finnish Institute of High Performance Sport KIHU, Jyväskylä, Finland; 3 Tampere Research Centre of Sports Medicine, UKK Institute for Health Promotion Research, Tampere, Finland; 4 Department of Pediatrics and Adolescent Medicine, Turku University Hospital, Turku, Finland; 5 Institute of Biomedicine, University of Turku and Clinical Microbiology Laboratory, Turku University Hospital, Turku, Finland; 6 Fimlab Laboratories, Tampere, Finland; 7 Faculty of Medicine and Health Technology, Tampere University, Tampere, Finland

**Keywords:** athlete, common cold, ice hockey, respiratory infection, respiratory virus

## Abstract

**Background:**

Little is known about viral acute respiratory infections (ARIs) in ice hockey teams. This study aimed to investigate the occurrence, etiology, and transmission of ARIs in three professional ice hockey teams playing the Finnish Elite League during the 2022–2023 season.

**Methods:**

A total of 81 players and 18 staff members were prospectively followed during a 7-month season. Nasal swabs for the detection of 16 respiratory viruses and rhinovirus typing were collected from all players at the onset of sore throat, rhinorrhea, nasal congestion, cough, and/or fever and, for one team, also once monthly from asymptomatic players. Viral diagnostics was done using the Allplex Respiratory Panels or FilmArray.

**Results:**

Ninety-three percent (75/81) of the players experienced at least one ARI, and 17% (14/81) experienced three or more. A viral cause was detected in 65% of the 129 cases, with 23 different viruses. The most commonly detected viruses were SARS-CoV-2, rhinoviruses, and influenza A viruses. Thirteen potential cluster groupings (caused by 11 different viruses) of respiratory viral infections were detected, four of which were asymptomatic. Of the 104 illness episodes among the players for which symptom forms were completed, 25 (24%) were febrile. Thirty-six percent (37/104) of the players played while ill. Thirty-four percent (35/104) of the symptomatic players missed at least one game.

**Conclusion:**

Most professional ice hockey players suffered from viral ARI during the competitive season. Infections were contracted from both the community and the team. The clinical manifestations were mild or moderate, but the time loss from ARIs was evident.

## 1 Introduction

Acute respiratory infections (ARIs) are the most frequent cause of illness in athletes (1, 2), and most of them are of viral etiology (3). The clinical manifestation is usually a mild common cold, characterized by a sore throat, sneezing, rhinorrhea, nasal congestion, or a cough. Symptoms typically last 3–7 days (4, 5). Despite only causing a mainly mild disease, an ARI can lead to time loss in training and competitions (1, 6, 7).

In ice hockey players, in addition to heavy physical exercise, psychological stress, and frequent travelling (8), there are many behavioral factors that are likely to increase the risk of viral ARIs. These include the high frequency and intensity of close contact both in the locker room and on the ice, as well as heavy breathing during play (4, 9, 10). However, only a limited number of studies have addressed ARIs in ice hockey players (11–13). Two earlier studies found that influenza A virus and SARS-CoV-2 readily spread within teams and also to opposing teams (14, 15). In a recent ARI outbreak within a professional ice hockey team, only a few of the many respiratory viral infections were spread between players, suggesting an important transmission from the community (16).

Further research is needed to understand viral ARIs among ice hockey players in order to develop preventive measures. Consequently, we investigated the occurrence, etiology, and transmission of ARIs in three professional ice hockey teams playing in the Finnish Elite League during the 2022–2023 season. We also describe the time loss from ARIs in players by reporting the games and practices missed.

## 2 Materials and methods

### 2.1 Study planning and participants

This prospective observational study was carried out during the 2022–2023 season of the Finnish Elite League. Monitoring of ARIs was provided for three professional ice hockey teams. The recruitment period for this study was between August 1 and 31, 2022.

All players and staff were instructed to report symptoms of an ARI immediately to the team physician or physiotherapist. At the onset of an ARI, a nasal mucus specimen was collected at a depth of 3–4 cm using a flocked nasal swab (553C, Copan, Brescia, Italy). Swabs from Team I were placed in Copan UTM RT 3 mL tubes, and swabs from Teams II and III were placed in dry tubes. In Teams I and II, the swabs were collected by the team physician or another healthcare professional. Team III players self-collected nasal swabs because they also collected swabs once monthly while asymptomatic, starting in October. They received verbal instructions to insert a flocked nasal swab to a depth of 3–4 cm (17, 18).

This study complied with the Declaration of Helsinki as revised in 2000 and all study-related activities were conducted according to Good Clinical Practice. The study protocol was approved by the Ethics Committee of the Hospital District of Southwest Finland (41/1801/2022). Written informed consent was obtained from all study subjects.

### 2.2 Assessment of illness

ARI was defined as the acute onset of at least one of the following symptoms, lasting for at least 1 day: a sore throat, rhinorrhea, nasal congestion, a cough, or a fever (ear or axillary temperature ≥37.8 °C). Symptoms were documented on a standardized form once during the first 3 days of illness (19). A four-point severity scale (0 = absent, 1 = mild, 2 = moderate, and 3 = severe) was used, with the exception of fever (20). ARI was classified as moderate if the player had at least one moderate symptom, and as severe if the player had at least one severe symptom.

Asymptomatic viral ARIs were defined as the detection of a respiratory virus in participants who did not exhibit respiratory symptoms (21).

### 2.3 Microbiological studies

Laboratory testing was conducted locally in Tampere, Jyväskylä, and Turku. In Tampere and Turku, the analyses were performed in accredited test laboratories, and in Jyväskylä using a private machine located at the facilities of the Finnish Institute of High Performance KIHU. For samples from Teams I and III, the Allplex Respiratory Panels 1–3 (Seegene, Seoul, South Korea) were used according to the manufacturer's instructions to detect the following viruses: respiratory syncytial virus (RSV) A and B, adenovirus (AdV), influenza A (Inf A) (subtyping H1, H1pdm09, and H3) and B viruses (Inf B), rhinovirus (RV), enteroviruses (EV), parainfluenza type 1–4 viruses (PIV1–4), human coronaviruses (HCoV) 229E, OC43, and NL63, human bocavirus (HBoV), and human metapneumovirus (MPV). Severe acute respiratory syndrome coronavirus 2 (SARS-CoV-2) was detected using cobas® SARS-CoV-2 (Roche Cobas 8800) (Roche Diagnostics, Rotkreuz, Switzerland) or a laboratory designed test (22).

For the analysis of Team II samples, the FilmArray Respiratory Panel 2 plus (BioFire, Salt Lake City, UT, USA), an automated point-of-care test (POCT), was used. This panel detects RSV (without subtyping), AdV, Inf A (subtypes H1, H1pdm09, and H3) and B, RV/EV (without specifying which), PIV1–4, HCoV-229E, -OC43, -HKU1, and -NL63, MPV, and SARS-CoV-2.

Following the initial analyses, Team II samples that tested positive for RV/EV were sent to Turku University Hospital virus laboratory, where RVs and EVs were differentiated and typed using VP4/2 gene sequencing (23). Only the samples from Team II were typed because the FilmArray panel does not specify RV or EV.

A potential viral cluster was defined as three detections of the same virus within a 2-week period. This was considered an unusual aggregation event.

### 2.4 Carbon dioxide monitoring in the locker room

The carbon dioxide concentration in the locker room was monitored during one ice hockey game in each team's home arena. Measurements were purchased from the University of Applied Sciences, Turku, Finland. Carbon dioxide served as an indicator of occupant-emitted contaminant levels and ventilation adequacy (24). The measurements were performed using the Miran DLS device, which has a measurement range of 0–5,000 parts-per-million (ppm) and an accuracy of ±30 ppm or ±3% of the reading. In Tampere, Jyväskylä, and Turku, six, nine, and five devices were installed in the locker room, respectively. The devices were positioned for instance on top of shelves. They were not placed directly in the airflow from the supply air terminal unit. Monitoring began before the players arrived at the arena and concluded after their departure, lasting 4–5 h.

According to the Classification of Indoor Environment 2018 (25), the target value for a “Good Indoor Environment” is below 950 ppm and the target value for a “Satisfactory Indoor Environment” is below 1,200 ppm. The limit values include the outdoor air concentration (approximately 400 ppm). The limit values are not related to health risks caused by carbon dioxide; they are related to the amount of ventilation, which can be used to determine the efficacy of the ventilation in removing human respiratory effluents from indoor environments.

### 2.5 Statistics

Normally distributed data are presented as mean (SD), and non-normally distributed/skewed data are presented as median [interquartile range (IQR)].

## 3 Results

Team I had 35 players, Team II 31 players, and Team III 25 players. Ten players from Team III refused to participate. In total, 81 players were included in the study. The mean (SD) age of the players was 26 (4) years in Team I, 24 (4) years in Team II, and 26 (5) years in Team III. In addition, the study included 11 staff members from Team I and seven from Team II; the staff members of Team III were not willing to participate in the study.

The players and staff members participated in the monitoring starting on September 1, 2022. In Finland, the regular season games begin in September and are evenly distributed until the start of the playoffs. Team II did not advance to the playoffs; therefore, monitoring concluded on March 11, 2023. Teams I and III progressed to the playoffs, with monitoring concluding on April 20, 2023, and March 16, 2023, respectively. Consequently, Teams I, II, and III were monitored for a total of 33, 27, and 28 weeks (mean 29 weeks), respectively. During the season, the teams played a total of 72, 60, and 63 games (mean 65 games), respectively.

### 3.1 Occurrence of symptomatic ARIs

In Team I, a total of 50 symptomatic ARIs were reported among the players, with 33 (94%) of the 35 players experiencing at least one ARI (Table 1). Ten players had two ARIs, two players had three ARIs, and one player had four ARIs. In Team II, 62 symptomatic ARIs were reported among the players, affecting 30 (97%) of the 31 players. Eleven players had two ARIs, six players had three ARIs, and three players had four ARIs. In Team III, 17 symptomatic ARIs were reported among the players, with 12 (80%) of the 15 players experiencing at least one ARI. One player had two ARIs, and two players had three ARIs.

**Table 1:** Symptomatic ARIs in players during the 7-month study period.

Team	Players	Symptomatic ARIs	Players with ≥1 ARI	Players with 2 ARIs	Players with 3 ARIs	Players with 4 ARIs
Team I	35	50	33 (94)	10 (29)	2 (6)	1 (3)
Team II	31	62	30 (97)	11 (35)	6 (19)	3 (10)
Team III	15	17	12 (80)	1 (7)	2 (13)	–
Total	81	129	75 (93)	22 (27)	10 (12)	4 (5)

Data are presented as *n* or *n* (%) unless otherwise indicated. ARI, acute respiratory infection.

In total, 129 symptomatic ARIs were reported among 75 (93%) of the 81 players during the 7-month competitive season. Two or more ARIs were identified in 44% of the players, and three or more ARIs in 17%. Additionally, a total of 23 symptomatic ARIs were reported among the staff members, with nine of the 11 staff members in Team I and all seven staff members in Team II experiencing at least one ARI. Four staff members experienced two ARIs, and one experienced four ARIs.

The highest incidence of symptomatic ARIs was observed from November to January, accounting for 61% of all symptomatic infections. The incidence was lowest in February (3%).

### 3.2 Etiology of ARIs

Viral etiology was detected in 84 (65%) out of 129 ARI episodes among the athletes. In professionally collected samples from Teams I and II, the etiology was identified in 76 (68%) out of 112 cases. In samples self-collected by players from Team III, the etiology was detected in eight (47%) out of 17 cases. One nasal mucus sample from Team I was lost in transportation. Twenty-three different respiratory viruses were identified (Table 2). The most commonly detected viruses were SARS-CoV-2 (*n* = 19, 21%), RVs (*n* = 18, 20%), and Inf A (*n* = 9, 11%). Three players had a co-infection of two viruses (MPV and RSV B; MPV and RV-C11; MPV and RV-C18) and one player had a co-infection of three viruses (RSV, RV-B48 and HCoV-OC43).

**Table 2:** Symptomatic respiratory viral infections in each team and across all teams combined.

Virus	Team I		Team II		Team III	All teams	
	Players	Staff	Players	Staff	Players	Players	Staff
Adenovirus	1				1	2	0
Coxsackie virus A6			1			1	0
Enterovirus D68			8			8	0
Human coronavirus HKU1			1			1	0
Human coronavirus NL63	1	1			1	2	1
Human coronavirus OC43	2		2			4	0
Human coronavirus 229E		1			1	1	1
Influenza virus A(H3)			7	1	1	8	1
Influenza virus A(pdm09)	1					1	0
Influenza virus B	2		2			4	0
Human metapneumovirus	2		4		1	7	0
Parainfluenza virus 1			2	1		2	1
Parainfluenza virus 3	3	1	1			4	1
Rhinovirus A23			1			1	0
Rhinovirus A81			1			1	0
Rhinovirus A101			2			2	0
Rhinovirus B48			3			3	0
Rhinovirus B91			1			1	0
Rhinovirus C11			1			1	0
Rhinovirus C15			1	2		1	2
Rhinovirus C18			2	1		2	1
Rhinovirus	3	1	2		1	6	1
Respiratory syncytial virus B	2	1			2	4	1
Respiratory syncytial virus			5	1		5	1
Severe acute respiratory syndrome coronavirus 2	15	7	4	1		19	8

Only Team II samples positive for RV/EV were typed by gene sequencing, as the FilmArray panel does not specify RV or EV. The FilmArray panel also does not subtype RSV into A or B.

Among the staff members of Teams I and II, the etiology of ARIs was detected in 18 (78%) out of 23 cases. Nine different respiratory viruses were identified (Table 2). One staff member had a co-infection of two viruses (PIV1 and RSV).

### 3.3 Occurrence of asymptomatic ARIs in the players of team III

Twenty-two (31%) of 70 samples from 15 asymptomatic players of Team III were virus positive. The monthly positivity rate ranged from 20% to 55%. Six different respiratory viruses were identified: RV 10, HCoV-NL63 5, HCoV-OC43 3, RSV B 3, HCoV-229E 2, and RSV A 1. Two players had a co-infection of two viruses (both had HCoV-NL63 and HCoV-OC43).

### 3.4 Transmission of ARI within teams; occurrence of potential viral clusters

#### 3.4.1 Team I

**Figure 1:**
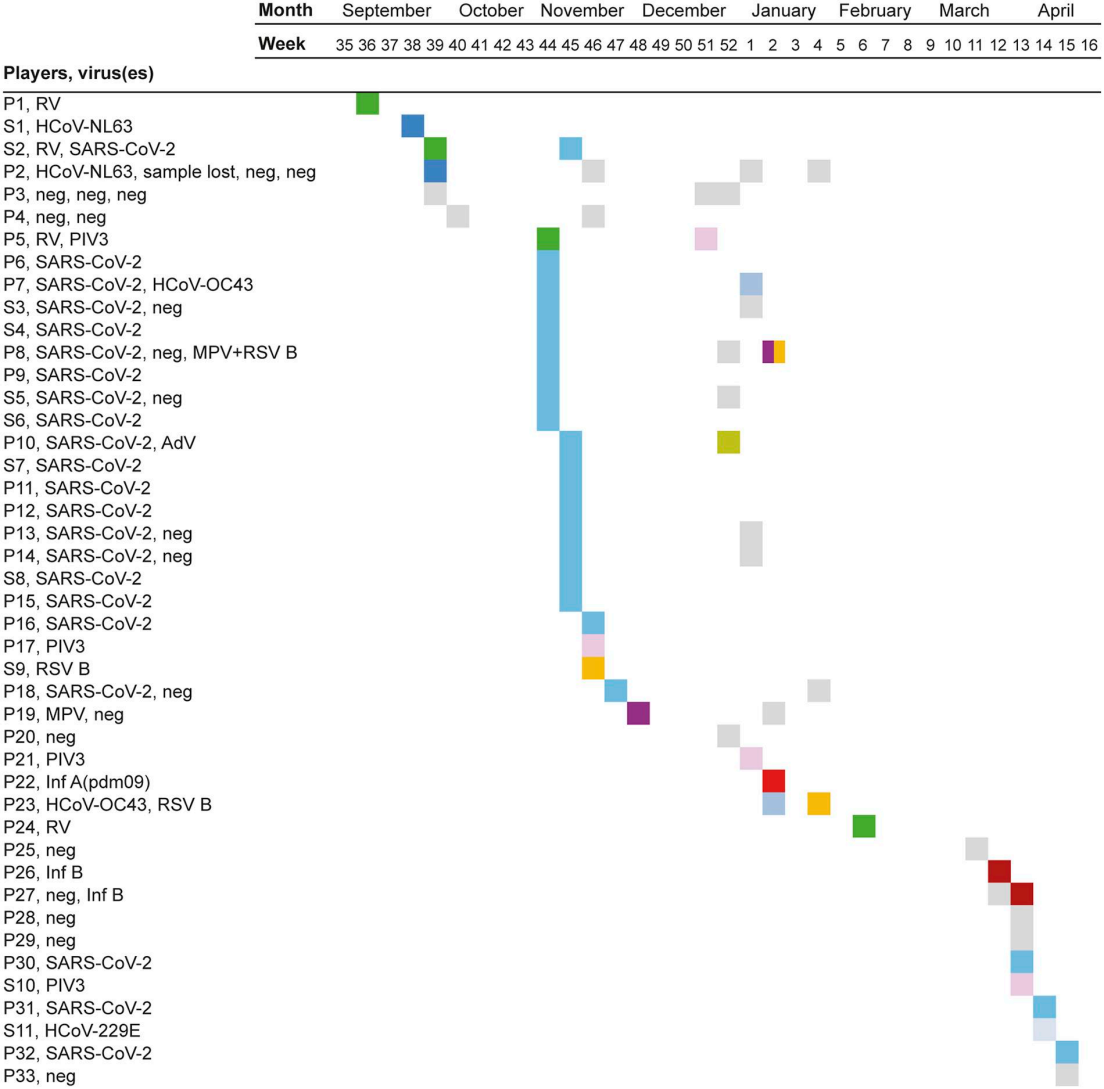

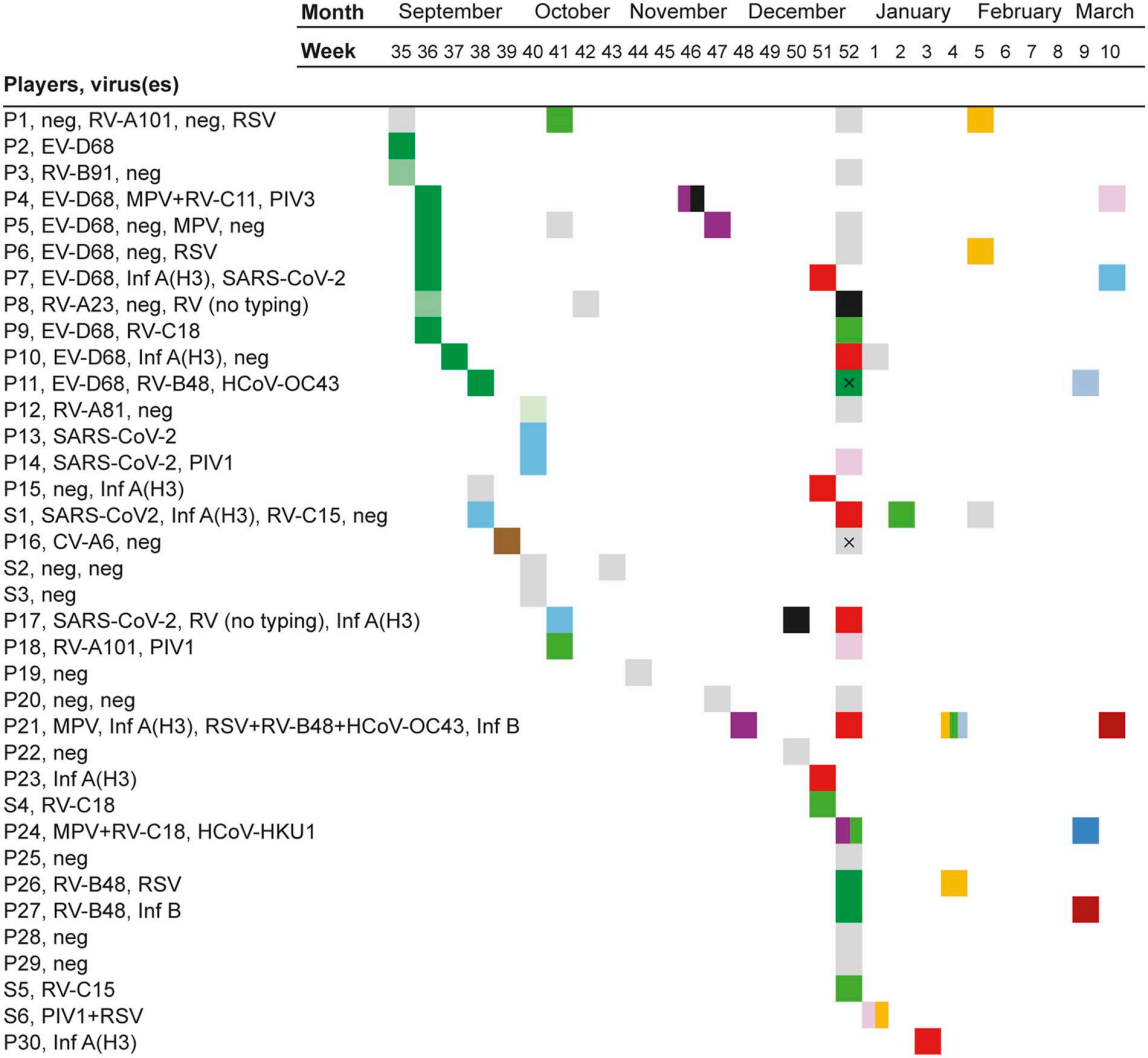

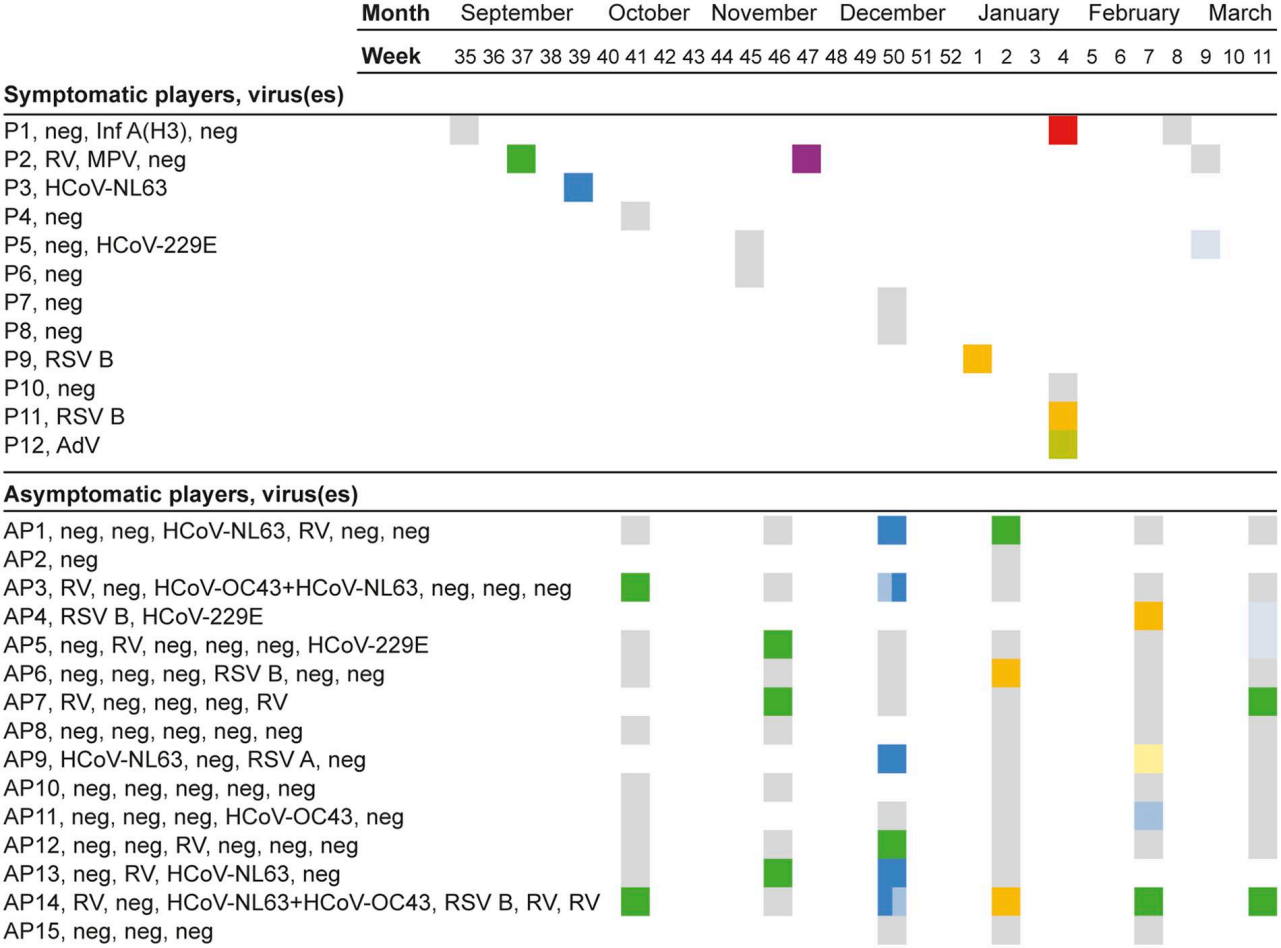
**(A–C)** Timeline of respiratory viral infections in teams I–III. P, player; AP, asymptomatic player; S, staff: 
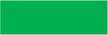
 RV, rhinovirus; 
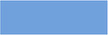
 HCoV-NL63, human coronavirus NL63; 
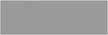
 neg, negative; 
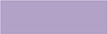
 SARS-CoV-2, severe acute respiratory syndrome coronavirus 2; 
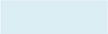
 PIV3, parainfluenza type 3 virus; 
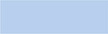
 HCoV-OC43, human coronavirus OC43; 
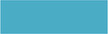
 MPV, metapneumovirus; 
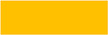
 RSV B, respiratory syncytial virus B; 
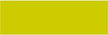
 AdV, adenovirus; 
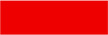
 Inf A, influenza A virus; 
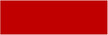
 Inf B, influenza B virus; 
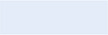
 HCoV-229E, human coronavirus 229E; 
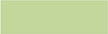
 RV-A101, rhinovirus A101; 
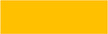
 RSV, respiratory syncytial virus; 
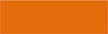
 EV, enterovirus; 
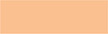
 RV-B91, rhinovirus B91; 
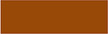
 RV-C11, rhinovirus C11; 
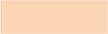
 RV-A23, rhinovirus A23; 
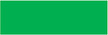
 RV-C18, rhinovirus C18; 
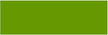
 RV-B48; rhinovirus B48; 
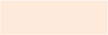
 RV-A81, rhinovirus A81; 
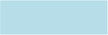
 PIV1, parainfluenza type 1 virus; 
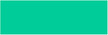
 RV-C15, rhinovirus C15; 
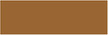
 CV-A6, coxsackievirus A6; 
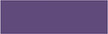
 HCoV-HKU1, human coronavirus HKU1; 
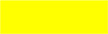
 RSV A, respiratory syncytial virus A. Color coding based on the virus finding indicates sample collection time. Test results for asymptomatic individuals in Team II are marked with an X. Distinct ARI episodes are separated by commas.

On 4 November 2022, SARS-CoV-2 was detected in two players (Figure 1A). During the following 6 days, SARS-CoV-2 was detected in eight players and seven staff members. The next COVID-19 cases were subsequently identified 10 (one player) and 21 (one player) days after the initial SARS-CoV-2 detection. During this cluster, SARS-CoV-2 was detected in 12 players and seven staff members.

On 27 March 2023, SARS-CoV-2 was detected in one player. The following cases were subsequently identified eight (one player) and 14 (one player) days after the initial detection. During this cluster, SARS-CoV-2 was detected in three players.

#### 3.4.2 Team II

On 1 September 2022, EV-D68 was detected in one player (Figure 1B). Five to eight days later, four players were diagnosed with EV-D68. The next EV-D68 cases were subsequently identified 12 (one player) and 20 (one player) days after the initial EV-D68 detection. During this cluster, EV-D68 was detected in eight players.

On 23 September 2022, SARS-CoV-2 was detected in one staff member. Subsequent cases were identified in players 10, 11 and 17 days later. During this cluster, SARS-CoV-2 was found in one staff member and three players.

On 16 November 2022, MPV was detected in one player. Additional cases were identified five and 13 days later, each in one player. During this potential cluster event, MPV was found in three players.

On 20 December 2022, influenza A subtype H3 was detected in one player. Subsequent cases were identified 1 day (one player), 2 days (one player), and 8 days later (three players and one staff member). During this potential cluster event, influenza A (H3) was found in six players and one staff member.

On 22 December 2022, RV was detected in one staff member. Subsequent cases were identified 6 and 7 days later. During this potential cluster event, RV was found in one staff member and two players. Genetic analysis showed RV-C18.

On 28 December 2022, parainfluenza type 1 virus was detected in two players. Ten days later, parainfluenza type 1 virus was detected in one staff member. During this potential cluster event, parainfluenza type 1 virus was detected in two players and one staff member.

On 28 December 2022, RV was detected in three players, one of whom was asymptomatic. Genetic analysis revealed RV-B48.

On 27 January 2023, RSV was detected in one player. Subsequent cases were identified 2 and 3 days later. During this potential cluster event, RSV was found in four players. The FilmArray panel did not subtype RSV.

#### 3.4.3 Team III

On 15 November 2022, RV was detected in three samples from asymptomatic players (Figure 1C).

On 11 December 2022, HCoV-NL63 was detected in five asymptomatic players.

On 2 January 2023, RSV B was detected in one symptomatic player. Thirteen days later, RSV B was detected in two asymptomatic players. Eight days after that, RSV B was detected in another symptomatic player. During this potential cluster event, RSV B was identified in two symptomatic and two asymptomatic players.

### 3.5 Clinical manifestations of the ARIs

Symptom forms were completed for 104 of the 129 symptomatic ARI cases among the athletes. Symptoms were mostly mild or moderate: 64% (67/104) of players had neither fever nor severe symptoms. Twenty-five febrile illness episodes (24%, 25/104) were reported (Inf A 6, RV 3, EV-D68 2, Inf B 2, PIV3 2, SARS-CoV-2 1, HCoV-OC43 1, RSV B 1, AdV 1, RSV B + MPV 1, RSV + RV-B48 + OC43 1, neg 4). A sore throat was reported by 74% (77/104) of players, nasal congestion by 73% (76/104), a cough by 65% (68/104), and rhinorrhea by 62% (64/104). The median symptom duration was 4.5 days (IQR 3–7, range 0–38). Among those with fever, the median symptom duration was 7 days (IQR 4–9, range 2–38). Fifteen ARI episodes (14%) lasted more than 7 days.

Among the eight players with EV-D68, the median symptom duration was 10.5 days (IQR 4–14, range 2–30). Five players with EV-D68 had a sore throat, rhinorrhea, and nasal congestion, and three players had a cough. Two players suffered from febrile illness, one of whom reported a severe cough.

### 3.6 Time loss from ARIs

Overall, 34% (35/104) of symptomatic players missed at least one game, and 52% (54/104) missed at least one practice. The median number of games and practices missed by players due to an ARI was 0 games (IQR 0–1, range 0–4) and 2 practices (IQR 0–4, range 0–10), respectively. At the time of the first SARS-CoV-2 cluster in Team I, the Finnish Hockey League was on a break due to national team games. As there were no league games during this period, nine out of the 11 infected players did not miss any games. However, one player was unable to join the national team. The players of Team I who contracted COVID-19 gradually returned to training over a period of 10 days. Three players with EV-D68 infection continued to train and play despite being infected.

### 3.7 Carbon dioxide concentration in the locker room

**Figure 2:**
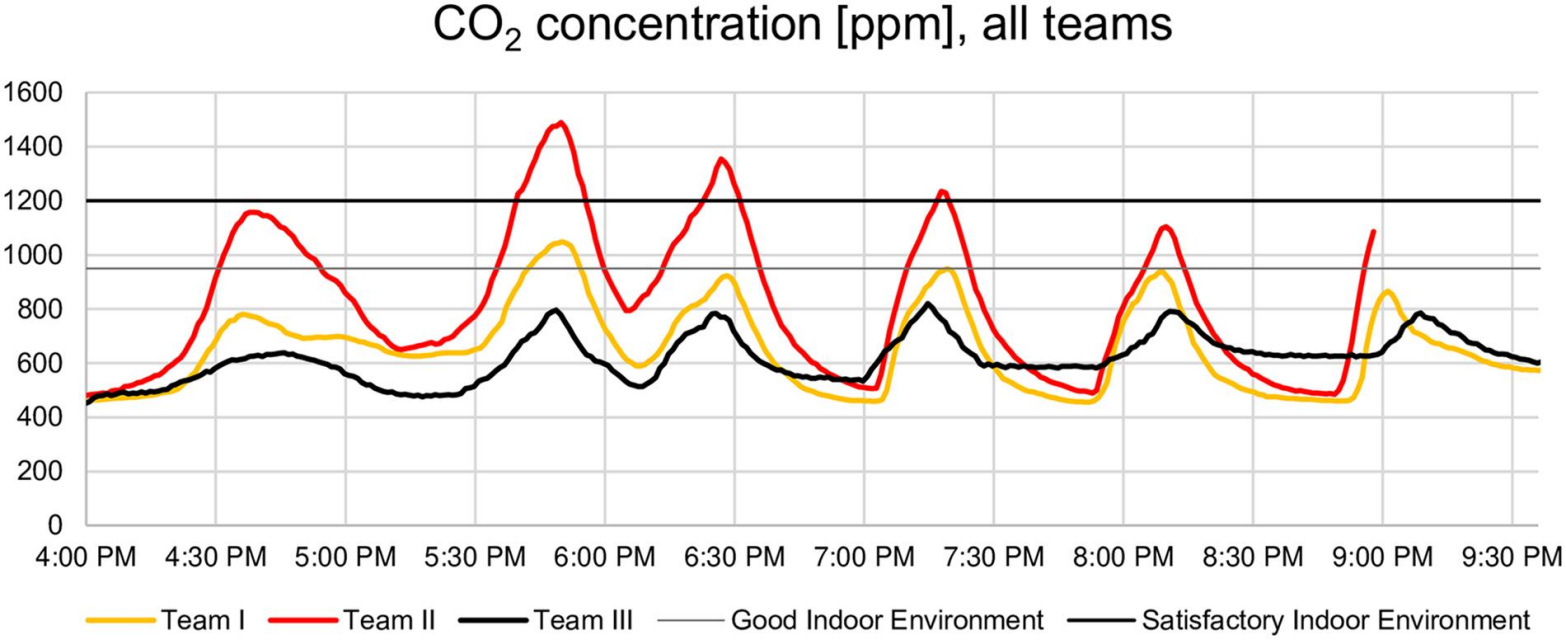
The carbon dioxide (CO_2_) concentration in the locker room during one ice hockey game per team. CO_2_ measurements began prior to player arrival and concluded after all players had left the arena. Each game started at 6:30 p.m. and was preceded by a pre-game warm-up. The games for Teams I, II, and III ended at 8:56 p.m., 8:45 p.m., and 8:49 p.m., respectively. The first CO_2_ peak coincided with the players’ initial arrival. The second peak occurred when players returned to put on their gear before the warm-up, the third followed the warm-up session, the fourth and fifth corresponded to intermissions, and the sixth occurred after the game when players returned to the locker room before leaving the arena. Ppm, parts-per-million.

The carbon dioxide measurements revealed the occupied periods in the locker rooms, such as during intermissions in all the monitored locker rooms (Figure 2). During the hockey game, the average measurement curve remained within the target value for the “Good Indoor Environment” category in the arenas of Teams I and III (carbon dioxide concentration ranged from 441 to 1,048 ppm and from 425 to 820 ppm). In the arena of Team II, the average curve exceeded the target value for the “Satisfactory Indoor Environment” during the first interval (maximum carbon dioxide concentration 1,354 ppm). In this arena, the average carbon dioxide concentration also exceeded the target value for the “Satisfactory Indoor Environment” category before the start of the game (maximum carbon dioxide concentration 1,488 ppm).

### 3.8 Family members

Ten players had young children (two, four, and four in Teams I, II, and III, respectively), who are a constant reservoir of respiratory viruses. In six (43%) of the 14 cases, the player suspected his children as the source of the infection.

## 4 Discussion

We found that 93% of the ice hockey players and 89% of the staff members experienced at least one ARI during the 7-month competitive season. Nearly half of the players and nearly one-third of the staff members experienced two or more ARIs. The viral cause of the infections was identified in 65% of the symptomatic players and 78% of the symptomatic staff members, with 23 different respiratory viruses detected. The most commonly detected viruses were SARS-CoV-2 (*n* = 27, 26%; players *n* = 19, staff *n* = 8), RVs (*n* = 22, 22%; players *n* = 18, staff *n* = 4), and Inf A (*n* = 10, 10%; players *n* = 9, staff *n* = 1). In 8% of symptomatic cases (9 out of 107), the virus likely transmitted within the team, resulting in potential infection clusters. These findings suggest that most infections were acquired from the community. The time loss from viral ARIs was marked. However, one-third of the players with ARI continued to play despite having symptoms.

Our finding that there were 1.6 ARI episodes during the 7-month study period is consistent with previous studies in ice hockey players. Overall, an average of 1–2 ARI episodes per season or year was reported in studies from Poland (11), Norway (12), and Sweden (13). Notably, none of the previous studies examined the etiology of ARIs. Compared with the staff, who shared many risk factors with the players, the players had a similar risk of ARIs: the staff members experienced a mean of 1.3 ARIs during the 7-month study period.

During the COVID-19 pandemic, it was established that many respiratory viral infections are asymptomatic (26). To date, there is little information on the occurrence of asymptomatic respiratory viral infections in athletes. In our previous study, 8% (2/26) of the skiers were found to have an asymptomatic rhinovirus infection during a 2-week winter sports competition (3). Similarly, another study involving elite skiers and orienteers reported that 8% (51/623) of samples collected during asymptomatic phases tested positive for respiratory viruses (5). A retrospective study of elite aquatic athletes diagnosed with COVID-19 found that 17% (68/401) remained asymptomatic (27). In the present study, a virus was detected in 31% (22/70) of the monthly routine screening samples collected from the asymptomatic players, most commonly rhinoviruses or seasonal coronaviruses. It is obvious that there are asymptomatic virus positive players all the time in the ice hockey team and asymptomatic viral ARIs may be an important source of infection (28).

Our data suggest that most infections were acquired from the community, with some occurring within the team. SARS-CoV-2 possibly spread within Team I on two occasions, while eight different respiratory viruses [EV-D68, SARS-CoV-2, MPV, Inf A (H3), RV-C18, PIV1, RV-B48, and RSV] and three different respiratory viruses (RV, HCoV-NL63, and RSV B) possibly spread within Teams II and III, respectively. The clusters were not established by genome sequencing and some may have been induced by different subtypes derived from the community. We previously reported influenza A outbreaks in two professional hockey teams that occurred shortly after they played two games against each other. Influenza A was diagnosed in 36% (9/25) of the players and in 15% (4/27) of the players, respectively (15). Another previous study conducted on hockey players suggested that a single asymptomatic COVID-19 carrier infected 79% (22/28) of his team's players (14). In another professional ice hockey team, only influenza A and HCoV-NL63 were possibly spread during the 40 days, suggesting an important contraction from the community (16).

ARIs can result in time loss from training and competition. A study of elite ice hockey players in the Norwegian professional league found that 60% (280/468) of all illness-related lost training time and competition days were due to respiratory illnesses (29). In this study, 34% (35/104) of the symptomatic players missed at least one game due to an ARI, and 52% (54/104) missed at least one practice, with a median of 0 games and 2 practices missed per player, respectively. During the abbreviated 2020–21 season, National Hockey League (NHL) players missed an average of 5.6 games due to COVID-19 (30). In team sports, the disease burden of febrile ARIs can be substantial for the team during an outbreak (16). During outbreaks of influenza A in two professional ice hockey teams, seven players missed an important game (15).

In this study, 36% (37/104) of the players played despite having symptomatic ARI. In September 2022, several players from Team II tested positive for EV/RV. Due to the EV-D68 outbreak in Finland (23), the samples were retrospectively screened for EV-D68, revealing a cluster of EV-D68 infections within Team II. This was a surprising finding, as EV-D68 is predominantly detected in children under the age of five (31). EV-D68 is considered a potentially more harmful virus due to its association with acute flaccid myelitis (AFM) (31). Three players with mild/moderate symptoms continued to play, and no adverse events were reported. Additionally, 19 players experienced symptomatic COVID-19 during this study period; however, none developed long COVID (32). Of the 104 illness episodes for which symptom forms were completed in this study, 24% were febrile. In the NHL, to our knowledge, only fever sanctions an exemption from playing. Currently, there is insufficient evidence regarding the health risks of exercising while ill (6, 33). It is of note that infections with SARS-CoV-2, adenovirus, enteroviruses, and influenza viruses may be associated with myocarditis.

One potential risk factor for the transmission of viral ARIs among ice hockey players may be inadequate locker room ventilation, as indicated by elevated CO_2_ levels. A study of dormitory rooms in China found a direct association between high CO_2_ concentrations and common cold symptoms (34). In an analysis of air samples from community settings, respiratory pathogen genomic material presence and concentration were positively associated with CO_2_ concentration (35). Improving ventilation and indoor air quality reduces the risk of viral transmission in shared indoor environments (36). In indoor spaces like locker rooms, ventilation (filtration) can be enhanced by installing high-efficiency particulate air (HEPA) cleaners (36). To our knowledge, this preliminary study, although only a snapshot, is the first to monitor CO_2_ levels in locker rooms. Further research is required to determine whether improving air quality in locker rooms can mitigate the transmission of viral ARIs among hockey players.

Our study has several limitations. It was an uncontrolled observational study, and we cannot answer the question whether ice hockey players have an increased incidence of viral ARIs compared with the normal population. The number of players in our study remained small. From Team III, only 60% of the players were willing to participate in this study. Professional ice hockey players are a challenging target for a disciplined clinical study, as they are highly aware of their bodies and sensitive to interventions. The monthly collection of nasal swabs from Team III players may have caused some concern; however, this approach allowed us to estimate the proportion of players with asymptomatic ARIs. Nasal samples from Teams I and II were collected professionally, while players from Team III self-collected their samples. This may have contributed to the quality of the mucus samples and the fact that the etiology was identified in only 47% of cases in Team III, whereas it was identified in 68% of cases in Teams I and II. Samples from Team I were collected using different transport tubes than those from Teams II and III, which may have further affected the comparison of viral detections in different teams. The methods for viral diagnostics varied; however, we think this should not affect the overall results (3). We had no genomic sequencing evidence of the spread of respiratory viruses within the teams. We did not ask about vaccination status for influenza and COVID-19 because vaccine hesitancy may be more common in athletes than in the general population, and we did not want to approach this sensitive topic (37). We also do not have data on how many players with influenza were treated with antivirals. Symptom diaries were available for only 104 of the 129 cases, and six of the completed diaries lacked information on the onset and resolution of symptoms.

## 5 Conclusion

To the best of our knowledge, this is the first follow-up study to report on the etiology, transmission, and time loss from viral ARIs in a cohort of professional ice hockey players. A vast majority of professional ice hockey players suffered from viral ARIs during the competitive season. Infections were contracted from both the community and from the team. The clinical manifestations were mostly mild or moderate, but the time loss from ARIs was marked. Our observations suggest considering pharmaceutical and non-pharmaceutical prevention strategies for ice hockey teams during viral highs in the community. Pharmaceutical interventions include vaccinations (for influenza, COVID-19, and RSV) and the use of antivirals (oseltamivir or baloxavir) for virologically confirmed influenza, both of which are cost-effective. Major non-pharmaceutical interventions to prevent and control viral ARIs were well recognized during the COVID-19 pandemic; hand hygiene, face masks in public setting and during travelling, physical distancing, and isolation of people who are acutely ill (38). Adequate ventilation of indoor spaces is crucial. In addition, the use of air purifiers (HEPA filters) in locker rooms may reduce the transmission of viral ARIs (16, 39). These interventions are not always easy to implement in sports teams; however, they should be accepted by both the athletes and the staff.

## Data Availability

The original contributions presented in the study are included in the article/Supplementary Material, further inquiries can be directed to the corresponding author.
